# Rates and causes of neonatal and under-five mortality: insights from a national household survey in Pakistan

**DOI:** 10.7189/jogh.16.04211

**Published:** 2026-09-11

**Authors:** Asma Abdul Malik, Shabina Ariff, Muhammad Umer, Shanila Nooruddin, Muhammad Atif Habib, Arjumand Rizvi, Imran A Chauhadry, Sajid Bashir Soofi, Henry D Kalter, Robert E Black, Zulfiqar A Bhutta

**Affiliations:** 1 Institute for Global Health & Development, Aga Khan University, Karachi, Pakistan; 2 Centre of Excellence in Women and Child Health, Aga Khan University, Karachi, Pakistan; 3 Department of Paediatrics & Child Health, Aga Khan University, Karachi, Pakistan; 4 Institute for International Programs, Bloomberg School of Public Health, Johns Hopkins University, Baltimore, Maryland, USA; 5 Centre for Global Child Health, The Hospital for Sick Children, Toronto, Ontario, Canada

**Keywords:** neonatal mortality, under-five mortality, verbal and social autopsy, Pakistan, public health, cause-specific mortality

## Abstract

**Background:**

Neonatal and under-five mortality rates are critical indicators of a country’s health status and progress toward Sustainable Development Goals. Despite a decline in mortality rates globally, preventable child deaths continue to occur, particularly in Pakistan, which has one of the highest child mortality rates in the world. In this analysis, we present the latest cause-specific neonatal and under-five mortality estimates determined through verbal and social autopsy (VASA) procedures in a population-based household survey across Pakistan.

**Methods:**

We used a household survey based on the Pakistan National Nutrition Survey sampling frame, which covered 115 600 households in both urban and rural areas. We identified 3880 neonatal and post-neonatal under-five deaths within the three years prior to the survey. The VASA interviews were conducted in households where deaths were reported. We determined the causes of death using the computerised InterVA-5, InSilicoVA, and Expert Algorithm VA methods and the physician-certified VASA method, utilising thorough reviews by verbal autopsy-trained paediatric and obstetric physicians.

**Results:**

Neonatal mortality was estimated at 39.8 per 1000 live births (95% confidence interval (CI) = 39.3–40.3), with higher rates in rural (40.8; 95% CI = 40.2–41.4) compared to urban areas (37.3; 95% CI = 36.5–38.1). Post-neonatal under-five mortality was 35.9 per 1000 live births (95% CI = 35.4–36.4) and was also higher in rural (36.8; 95% CI = 36.2–37.4) than in urban areas (33.6; 95% CI = 32.6–34.5). The major causes of neonatal death included preterm birth complications, birth asphyxia, and infectious diseases. For post-neonatal under-five mortality, the leading causes were infectious diseases, diarrheal disorders, and pneumonia.

**Conclusions:**

Our findings highlight the persistent burden and disparities in neonatal and under-five mortality across urban and rural areas in Pakistan. Targeted interventions are essential to address these preventable deaths and reduce mortality rates in high-risk populations.

Neonatal and under-five mortality trends are important indicators of a country’s health status and progress towards achieving the Sustainable Development Goals (SDGs) [1]. Neonatal mortality is defined as the death of a newborn within the first 28 days after birth [2], while under-five child mortality refers to the death of a child aged 0–59 months [3]. In 2022, an estimated 4.9 million under-five children died globally; 2.3 million of these deaths occurred among neonates and 2.6 million among children aged 1–59 months [4]. Approximately 6500 neonates die each day, accounting for 47% of the under-five deaths [5]. Around two-thirds of all neonatal deaths occur in the first seven days of life [6], with approximately one million dying in the first 24 hours.

While more children are surviving to their fifth birthday than ever before, with the under-five mortality rate decreasing by over half since 2000 (from 76 per 1000 live births in 2000 to 37 per 1000 live births in 2022), progress has slowed since 2015 [7]. Millions of children still die each year from preventable and treatable causes [8]. Sustainable Development Goal (SDG) 3.2 aims to end preventable deaths of newborns and children under-five by 2030, with the goal of reducing neonatal mortality to at least 12 per 1000 live births and under-five mortality to at least 25 per 1000 live births in all countries [9]. Without immediate action, over 64 countries may fall short of meeting the SDG target for neonatal mortality by 2030. Despite the global decline in child mortality rates, entrenched inequities persist; under-five mortality rates remain 13 times higher in low-income than in high-income countries, and the chances of survival plummet for children born into the most impoverished households or living in conflict-stricken settings [8].

The countries of Southern and Central Asia, second only to those of sub-Saharan Africa, bear the heaviest burden of neonatal mortality, with 21 deaths per 1000 live births and an under-five mortality of 37 deaths per 1000 live births [5,10]. According to the estimates from the United Nations Inter-agency Group for Child Mortality Estimation (UN IGME) for 2022, Pakistan has high child mortality rates with a neonatal mortality rate (NMR) of 39 deaths per 1000 live births [11,12] and an under-five mortality rate of 61 deaths per 1000 live births [13,14]. Pakistan has seen a decline in neonatal, infant, and under-five mortality rates, since the last demographic and health survey (2012–2013); however, one in every 14 Pakistani children still does not survive to their fifth birthday [15]. Most neonatal and under-five deaths can be prevented through appropriate care during pregnancy, delivery, and the postpartum period. However, challenges such as inadequate health care facilities, shortages of healthcare professionals, poor care-seeking behaviours, and high out-of-pocket costs are barriers to accessing these essential services [16,17]. Many low- and lower-middle-income countries have reduced their under-five mortality rate, demonstrating the effectiveness of both health facility-based and community-based interventions in significantly reducing preventable deaths, even in resource-constrained settings [18–20]. Given Pakistan’s slow progress in reducing neonatal mortality over the past decade, there is a pressing need to understand the epidemiology and causes of neonatal deaths to meet the SDG targets. While urban and rural disparities in neonatal and under-five mortality have been recognised, recent nationally representative, cause-specific mortality estimates are lacking. We address this gap by providing up-to-date population level estimates of neonatal and child mortality and evaluating the determinants using verbal and social autopsy (VASA) methods. In the absence of a comprehensive registration system, the verbal autopsy is a validated retrospective inquiry method for determining the causes of death through in-depth *post-mortem* interviews with primary caregivers [21,22] and has been established as a valuable tool in resource-limited settings [23]. Our team at the Aga Khan University (AKU), in collaboration with Johns Hopkins University (JHU), conducted a population-based household survey in Pakistan, aiming to estimate the cause-specific neonatal and under-five mortality rates in Pakistan using VASA methods. The goal was to generate population-level estimates at the community level, capture deaths both within and outside healthcare facilities, and identify contextual factors, care-seeking delays, and gaps in service delivery to provide actionable insights for strengthening maternal and child health interventions in high-burden settings.

## METHODS

We performed a household survey using the sampling frame of the Pakistan National Nutrition Survey (PNNS) provided to the AKU by the Pakistan Bureau of Statistics [24]. The PNNS employed a stratified, multi-stage cluster sampling strategy to ensure representativeness across four provinces (Punjab, Sindh, Balochistan, and Khyber Pakhtunkhwa), the Islamabad Capital Territory (ICT), and the two administrative regions of Azad Jammu & Kashmir and Gilgit Baltistan, covering all 156 districts of Pakistan. Clusters were systematically defined based on enumeration blocks for urban and villages for rural areas, after which households were randomly selected within each cluster. Subsequently, VASA interviews were conducted in the households where neonatal and post-neonatal under-five deaths were identified within the last three years preceding the survey. Full methodological details, including sampling, questionnaire adaptation, training, and analytical procedures, are reported in the accompanying study protocol [25].

### Sampling

#### Household selection for mortality rates and VASA interview

The PNNS covered urban and rural areas across four provinces (Sindh, Punjab, Baluchistan, and Khyber Pakhtunkhwa) of Pakistan, the Islamabad Capital Territory (ICT), the two administrative regions of Azad Jammu and Kashmir, and Gilgit Baltistan. It took place between April 2018 and February 2019 and covered all 156 districts of Pakistan, totalling 115,600 households. The PNNS sampling frame served as the basis for a separate, larger mortality survey, which our team conducted across 4,475 clusters covering 3.4 million individuals in 502,933 households. This mortality survey (distinct from the NNS itself) collected data on all neonatal and post-neonatal under-five deaths occurring in the three years preceding the survey, through a line listing activity. Subsequently, and distinct from both the PNNS and the mortality survey, our team conducted VASA interviews exclusively in households where deaths had been identified through the mortality survey. For the VASA interviews, we calculated that we would require a sample size of 4000 deaths (including 2100 neonatal and 2100 post-neonatal under-five deaths) to ascertain the cause of death with 3% precision, assuming a 50% proportion, further inflation by 1.5 for design effect, and a 15% non-response rate. The VASA interviews were conducted from October 2018 to July 2019 and distributed across each province, ICT, and the two administrative areas based on the proportionate population size. We used a computer-generated programme to randomly select households where deaths were identified. The VASA questionnaire was completed for neonatal deaths and post-neonatal under-five deaths.

#### Cause of death – VASA questionnaire

We used the VASA questionnaire, which combines the WHO’s Verbal Autopsy 2016 questionnaire and the Johns Hopkins Bloomberg School of Public Health/Institute for International Programs (JHU/IIP) Social Autopsy (SA) tool [26,27] and includes modules designed for assessing neonatal and post-neonatal under-five deaths, and recording demographic information, pregnancy and delivery circumstances, and symptoms preceding death. The VASA tool was updated to standardise indicators, question wording, and formatting, emphasising the evaluation of under-five deaths and including assessments of preventive and curative factors [26]. Trained field teams conducted VASA interviews using a computer-assisted personal interviewing method through handheld devices. The VASA questionnaire was translated into Urdu, Pakistan’s primary language, and back translated to English to ensure language compatibility and consistency [28].

### Study outcomes

The primary outcomes of this study were the cause-specific neonatal and under-five mortality rates in Pakistan. Causes of death were determined using VASA methods executed through both computer-coded verbal autopsy (CCVA) and physician-certified verbal autopsy (PCVA) approaches based on standardised algorithms and expert review.

### Data collection

The VASA field teams consisted of a male supervisor and female interviewers who had undergone seven-day classroom training, including role-playing exercises and two days of practical field training. Senior AKU faculty facilitated five provincial-level workshops as part of this training program. Separately, a JHU VASA expert provided supplementary training to teams responsible for cause-of-death analysis, focusing on identifying causes of death using the ICD-10 [29]. Field supervisors monitored completed interviews in real time and revisited a random subset of completed interviews to verify that responses were consistent and data had been accurately recorded. Interviewer performance was evaluated based on adherence to standard protocols and accuracy in data entry. Households where neonatal or post-neonatal under-five deaths had been identified, caregivers were approached to explain the purpose of the study and obtain informed consent.

### Data analysis

We conducted descriptive analysis to examine the background characteristics of neonatal and post-neonatal under-five deaths and their causes. Mortality rates were calculated as deaths per 1,000 live births, with 95% confidence intervals (CIs). The average cause-of-death proportions was calculated as deaths per 1,000 live births, with 95% CIs across all three CCVA algorithms. The average cause-of-death proportions were calculated across all three CCVA algorithms, InterVA-5, InSilicoVA, and EAVA, to smooth variation across methods. All statistical analyses were performed using Stata, version 18 (StataCorp LLC., College Station, Texas, USA).

Given the reliance on caregiver-reported data, we implemented a multi-step validation process to address concerns about potential misclassification of causes of death. Two individuals from this trained team assessed each death independently, reviewing both the narrative and structured data from the VASA questionnaires, with any discrepancies in cause allocation reviewed and resolved through discussion with a third senior neonatal or obstetric specialist. In cases where consensus could not be reached among the three physicians, or where available information was insufficient to categorise the cause of death, the case was labelled as ‘undetermined’.

Alongside this PCVA process, CCVA methods were applied to provide a standardised, algorithm-based framework for cause-of-death attribution. The three CCVA algorithms used were InterVA-5 (Interpreting Verbal Autopsy, version 5) and InSilicoVA (In Silico Verbal Autopsy), which utilise probabilistic models to determine causes of death, and the Expert Algorithm Verbal Autopsy (EAVA), which relies on deterministic medical decision-making rules and expert-derived algorithms to identify the single most likely cause of death based on reported illness signs and symptoms [30–33]. CCVA labelled a cause of death as 'undetermined' when the VASA data were incomplete, conflicting, or lacked sufficient patterns to assign a probable cause. The AKU managed the data control system, overseeing the handling of the VASA database to ensure data quality.

## RESULTS

A total of 3880 VASA interviews were undertaken to investigate neonatal and post-neonatal under-five deaths in four provinces, ICT, and two administrative areas of Pakistan. Among these, 2074 (53.4%) pertained to neonatal deaths, while 1806 (46.5%) were for post-neonatal under-five deaths. Most of these deaths came from Punjab (n = 1322, 34.1%), followed by Sindh (n = 1098, 28.3%), Balochistan (n = 599, 15.4%), Khyber Pakhtunkhwa (n = 565, 14.6%), Gilgit Baltistan (n = 260, 6.7%), and Azad Jammu and Kashmir (n = 36, 0.9%). The average recall period was about one year (neonatal deaths: mean = 0.98, standard deviation (SD) = 1.0; post-neonatal under-five deaths: mean = 1.05, SD = 1.0). Mothers were the respondents for 91.5% of neonatal interviews and 89.8% of post-neonatal under-five interviews.

### Neonatal and under-five mortality rates

The NMR for 2017–2018 was 39.8 per 1000 live births (95% CI = 39.3–40.3), with rural areas exhibiting higher rates (40.8; 95% CI = 40.2–41.4) compared to urban areas (37.3; 95% CI = 36.5–38.1). The post-neonatal under-five mortality rate was 35.9 per 1000 live births (95% CI = 35.4–36.4), with higher rates in rural (36.8 per 1000 live births; 95% CI = 36.2–37.4) compared to the urban areas (33.6 per 1000 live births; 95% CI = 32.6–34.5). The NMR was highest in Khyber Pakhtunkhwa – Newly Merged Districts at 45.5 per 1000 live births (95% CI = 40.0–50.8), followed by Balochistan at 44.0 (95% CI = 43.0–45.1) and Sindh at 42.1 (95% CI = 40.8–43.3); it was lowest in Punjab at 36.5 (95% CI = 35.8–37.3) and ICT at 31.8 (95% CI = 28.2–35.3). The post-neonatal under-five mortality rate was highest in Azad Jammu and Kashmir at 48.0 (95% CI = 45.0–50.9), Gilgit Baltistan at 45.2 (95% CI = 43.1–47.3), and Balochistan at 37.3 (95% CI = 36.1–38.5), while it was lowest in KP-NMD at 26.0 (95% CI = 21.8–30.1), KP at 32.5 (95% CI = 31.2–33.8), and Sindh at 33.8 (95% CI = 32.3–35.3) (**Table 1**).

**Table 1:** Neonatal and post-neonatal under-five mortality rates in Pakistan

	Neonatal mortality rate (95% CI)*	Post-neonatal under-five mortality rate (95% CI)†
**National**	39.8 (39.3–40.3)	35.9 (35.4–36.4)
**Area**		
Rural	40.8 (40.2–41.4)	36.8 (36.2–37.4)
Urban	37.3 (36.5–38.1)	33.6 (32.6–34.5)
**Province/administrative area**		
KP-NMD	45.5 (40.0–50.8)	26.0 (21.8–30.1)
Balochistan	44.0 (43.0–45.1)	37.3 (36.1–38.5)
Sindh	42.1 (40.8–43.3)	33.8 (32.3–35.3)
Gilgit Baltistan	41.5 (39.5–43.4)	45.2 (43.1–47.3)
Azad Jammu & Kashmir	40.9 (38.1–43.7)	48.0 (45.0–50.9)
KP	40.4 (39.1–41.8)	32.5 (31.2–33.8)
Punjab	36.5 (35.8–37.3)	34.9 (34.1–35.7)
Islamabad	31.8 (28.2–35.3)	30.5 (26.0–34.8)

CI – confidence interval, KP – Khyber Pakhtunkhwa, KP-NMD – Khyber Pakhtunkhwa – Newly Merged Districts

*Deaths from age 0–28 days per 1000 live births.

†Deaths from age 29 days to 59 months per 1000 live births.

### Distribution of age and sex

The number of deaths was higher among males (n = 2,246, 57.9%) than females (n = 1,634, 42.1%) (Table S1 in the **Online Supplementary Document**), with the former accounting for 1,274 (61.4%) of the neonatal and 972 (53.8%) of post-neonatal child deaths. Nearly one-third (n = 632, 30.5%) of neonatal deaths occurred on the first day of life, with a higher proportion among males (n = 418, 32.8%) than females (n = 214, 26.8%) Early neonatal mortality, defined as death between 0–6 completed days, where 0 means being less than 24 hours, accounted for 1,702 (82.1%) neonatal deaths and 43.9% of the under-five mortality. Additionally, late neonatal deaths, defined as deaths between 7–27 days, accounted for 372 (18%) neonatal deaths and 372 (9.6%) under-five deaths. Post-neonatal under-five deaths accounted for 1,806 (46.5%) under-five deaths, while 1,042 (57.7%) under-five deaths occurred in infancy (1–11 months), accounting for 578 (59.5%) male and 464 (55.6%) female post-neonatal under-five deaths.

### Household and maternal characteristics

The average household density, defined as the number of individuals per room, was nearly four for both neonatal deaths (mean = 3.8, SD = 2.0) and post-neonatal under-five deaths (mean = 3.9, SD = 2.0).

Most of the mothers of deceased neonates (n = 1,356, 71.7%) and post-neonatal under-five children (n = 1,318, 81.3%) were over 24 years of age, with mean ages of 28.1 (SD = 6.4) years and 29.7 (SD = 6.2), respectively. Furthermore, 468 (23%) of the mothers of the deceased neonates received no antenatal care (ANC). Of those who sought ANC, 1,574 (97%) sought skilled providers, while 861 (42%) reported at least four ANC visits. Notably, only 87 (4.2%) of deceased neonates were born at term (37–40 weeks), while most (n = 1,706, 82.3%) were born moderate to late preterm. Additionally, 1,233 (60%) of mothers of deceased neonates reported no danger signs during pregnancy. Vaginal bleeding (n = 374, 18.0%), severe abdominal pain (n = 289, 13.9%), and convulsions (n = 216, 10.4%) were the most commonly reported danger signs. Additionally, 268 (25.8%) mothers reported having symptoms of pre-eclampsia or eclampsia, while 189 (18.2%) reported being anaemic (as told to them by their healthcare provider) during the last trimester of pregnancy (Table S2 in the **Online Supplementary Document**).

A total of 1,439 (70%) neonates were born at a health facility, while nearly one-third of deliveries (n = 622, 30%) were home births. Per the delivery data, skilled birth attendants attended 1,870 (90.2%) deliveries, while caesarean sections accounted for 440 (21%) of all deliveries of deceased neonates. Of the home births, only 197 (31.0%) reported using new or sterilised tools for cutting the umbilical cord during delivery, with 253 (40.0%) using clean or sterilised material for tying the cord. Among all births, around one-third (n = 628, 30.3%) of the neonates had antiseptic or antibiotic applied to the umbilical stump. More than half of the neonatal deaths (n = 1,194, 57.6%) occurred at a health facility, while 793 (38.2%) neonates passed away at home. Nearly one-third (n = 649, 36.0%) of the post-neonatal under-five children died at a health facility, while more than half (n = 987, 54.7%) passed away at home (Table S3 in the **Online Supplementary Document**). Overall, 456 (22.0%) women reported experiencing complications during labour. The complications included convulsions, high blood pressure, excessive bleeding, premature labour, obstructed labour, prolonged labour, uterine rupture, and retained placenta.

### Causes of neonatal mortality

Complications of preterm birth, birth asphyxia, and infectious diseases, including sepsis, meningitis, and pneumonia, emerged as the major causes of neonatal mortality in our study. However, the distribution of neonatal causes of death varied in different VASA analysis methods (**Table 2**). InterVA-5 identified preterm birth complications as the leading cause of death among neonates, accounting for 1,129 (54.4%) deaths, followed by birth asphyxia (n = 563, 27.1%) and infectious diseases (n=198, 9.8%). In contrast, InSilicoVA found that birth asphyxia was the most frequent cause of neonatal mortality (n = 891, 43.0%), followed by infectious diseases (n = 660, 31.8%) and preterm birth complications (n = 454, 21.9%). The EAVA method reported infectious diseases as the predominant cause (n = 703, 33.9%), while a significant proportion of deaths (n = 624, 30.1%) remained undetermined. The PCVA results showed infectious diseases as the most frequent cause of death (n = 738, 35.6%), followed by birth asphyxia (n = 683, 32.9%) and preterm birth complications (n = 343, 16.5%). The average across all three CCVA methods indicated birth asphyxia (29.5%) and preterm birth complications (28.7%) as the most common causes of death, followed by infectious diseases (25.1%). The inter-rater agreement between the two physicians assigning the cause of death was 46.57%, with a kappa statistic of 0.4102 indicating a satisfactory level of agreement [34].

**Table 2:** Neonatal causes of death by various verbal autopsy methods in Pakistan

Neonatal causes of death	InterVA5, n (%)	InSilicoVA, n (%)	EAVA, n (%)	Average %*	PCVA, n (%)
Birth asphyxia/intrapartum	563 (27.1)	891 (43.0)	379 (18.3)	29.5	683 (32.9)
Preterm birth complications	1,129 (54.4)	454 (21.9)	205 (9.9)	28.7	343 (16.5)
Infectious diseases (sepsis, meningitis, pneumonia)	198 (9.5)	660 (31.8)	703 (33.9)	25.1	738 (35.6)
*Sepsis/meningitis*	156 (7.5)	540 (26.0)	402 (19.4)	17.6	613 (29.6)
*Pneumonia*	42 (2.0)	120 (5.8)	301 (14.5)	7.4	125 (6.0)
Congenital malformation	103 (5.0)	16 (0.8)	101 (4.9)	3.5	77 (3.7)
Other unspecified causes†	0 (0.0)	41 (2.0)	15 (0.7)	0.9	0 (0.0)
Neonatal tetanus (NNT)	0 (0.0)	0 (0.0)	29 (1.4)	0.5	0 (0.0)
Diarrheal disorders	0 (0.0)	0 (0.0)	18 (0.9)	0.3	6 (0.3)
Accidents and injuries	0 (0.0)	11 (0.5)	0 (0.0)	0.2	11 (0.5)
Undetermined‡	81 (3.9)	1 (0.1)	624 (30.1)	11.3	216 (10.4)

CoD – cause of death, EAVA – expert algorithm verbal autopsy, InSilicoVA – InSilico verbal autopsy, InterVA-5 – interpreting verbal autopsy version 5, NNT – neonatal tetanus, PCVA – physician-certified verbal autopsy, VASA – verbal and social autopsy

*Average of InterVA5, InSilicoVA, and EAVA.

†Any death that does not meet any of the criteria for a diagnosis it is classified as unspecified.

‡When three physicians did not agree on the cause of death, it was classified as undetermined. Computer-coded VA labelled a cause of death as ‘undetermined’ when the verbal autopsy data are incomplete, conflicting, or lacks sufficient patterns to assign a probable cause.

### Causes of post-neonatal under-five mortality

Based on the average of the three CCVA methods, the predominant causes of mortality among children aged 1–59 months were other infectious diseases (29.5%), diarrheal diseases (19.2%), pneumonia (14.6%), injuries (7.2%), and malnutrition (4.9%). Other infectious diseases emerged as the leading cause of death across different methods, constituting 628 (34.8%) deaths per the InterVA-5, 566 (31.3%) per the InSilicoVA, 402 (22.3%) per the EAVA, and 337 (18.7%) per the PCVA. Diarrhoeal diseases contributed 395 (21.8%) deaths according to the InterVA-5, 363 (20.1%) according to the InSilicoVA, 280 (15.5%) according to EAVA, and 188 (10.4%) according to the PCVA. Pneumonia also stood out as a major cause of death, accounting for 103 (5.7%) deaths per the InterVA-5, 360 (19.9%) per the InSilicoVA, 329 (18.2%) per the EAVA, and 408 (22.6%) per the PCVA. Malnutrition was identified as a cause of death by all four methods, ranging from 82 (4.5%) deaths identified by the InterVA-5 to 158 (8.7%) by the PCVA. Other notable causes of death included injuries, which were likewise identified by all four methods, with the number of identified deaths ranging from 104 (5.8%) for the InterVA-5 to 146 (8.1%) for the InSilicoVA (**Table 3**). Both the physicians assigning the cause of under-five death agreed on 54.82% of the cases, with a kappa statistic of 0.4445 indicating a satisfactory level of agreement [34].

**Table 3:** Causes of death in children aged 1–59 months by various verbal autopsy methods in Pakistan

Cause of death among children 1–59 months of age	InterVA5, n (%)	InSilicoVA, n (%)	EAVA, n (%)	Average %*	PCVA, n (%)
Other infectious diseases (meningitis, encephalitis, sepsis, pertussis)	628 (34.8)	566 (31.3)	402 (22.3)	29.5	337 (18.7)
Diarrheal disorders	395 (21.9)	363 (20.1)	280 (15.5)	19.2	188 (10.4)
Pneumonia	103 (5.7)	360 (19.9)	329 (18.2)	14.6	408 (22.6)
Injuries	104 (5.8)	146 (8.1)	142 (7.9)	7.2	136 (7.5)
Malnutrition	82 (4.5)	102 (5.6)	81 (4.5)	4.9	158 (8.7)
Congenital malformation	94 (5.2)	52 (2.9)	34 (1.9)	3.3	1 (0.1)
Non communicable diseases	62 (3.4)	70 (3.9)	0 (0.0)	2.4	243 (13.5)
HIV	67 (3.7)	46 (2.5)	4 (0.2)	2.2	2 (0.1)
Measles	15 (0.8)	74 (4.1)	25 (1.4)	2.1	66 (3.7)
Malaria	27 (1.5)	15 (0.8)	33 (1.8)	1.4	2 (0.1)
Tuberculosis	3 (0.2)	4 (0.2)	0 (0.0)	0.1	16 (0.9)
Malignancy	2 (0.1)	3 (0.2)	0 (0.0)	0.1	44 (2.4)
Unspecified other cause of death†	20 (1.1)	3 (0.2)	124 (6.9)	2.3	71 (3.9)
Undetermined‡	204 (11.3)	2 (0.1)	352 (19.5)	10.3	134 (7.4)

CoD – cause of death, EAVA – expert algorithm verbal autopsy, HIV – human immunodeficiency virus, InSilicoVA – InSilico verbal autopsy, InterVA-5 – interpreting verbal autopsy version 5, NCDs – non-communicable diseases, PCVA – physician-certified verbal autopsy, VASA – verbal and social autopsy

*Average of InterVA5, InSilicoVA, and EAVA.

†Any death that does not meet any of the criteria for a diagnosis is classified as unspecified.

‡For PCVA, when three physicians did not agree on the cause of death, it was classified as undetermined. Computer-coded VA labelled a cause of death as ‘undetermined’ when the verbal autopsy data are incomplete, conflicting, or lacks sufficient patterns to assign a probable cause.

## DISCUSSION

This study provides insights into neonatal and under-five mortality patterns in Pakistan, emphasising contextual factors such as ANC utilisation, place of delivery, skilled birth attendance, and symptoms preceding death, using VASA methods. The national NMR from 2017–2018 stood at 39.8 per 1000 live births, which closely aligns with the NMR of 42.0 per 1000 live births reported in the Pakistan Demographic Health Survey 2018 [15], indicating an improved trend compared to the previously documented NMR of 46.0 per 1000 live births in 2015 [35]. The UN IGME recorded a relatively higher NMR of 44.0 per 1000 live births in 2017 [36]. We observed a post-neonatal under-five mortality rate of 35.9 per 1000 live births, which is not indicative of a substantial decrease from the 2015 estimate of 35.0 per 1000 live births [35]. In contrast, the UN IGME reported a post-neonatal under-five mortality rate of 31.0 per 1000 live births in 2017 [36]. These disparities in reported rates can be attributed to differences in data collection methods, sample selection, and reporting accuracy.

The gender disparity in NMR and post-neonatal under-five mortality rate we observed here, with males accounting for 57.9% and females for 42.1% of deaths, aligns with the estimates reported by the UN IGME and the Pakistan Demographic Health Survey [37]. The risk of mortality has been found to be 20% higher among male children during their perinatal period through infancy and childhood [38]. This sex-based difference in mortality rates may be attributed to biologically driven factors, particularly non-infectious causes of death such as prematurity, intrauterine growth retardation, and birth asphyxia [38]. Furthermore, the increased risk in male children could be associated with higher testosterone levels, which have been linked to suppressed immune function, and higher birth weights, predisposing them to delivery complications [38,39]. While biological vulnerabilities may play a role in this disparity, social determinants such as differential healthcare-seeking behaviour, feeding practices, and cultural preferences for male children in some settings may contribute to the observed patterns. Irrespective of the underlying reasons, these disparities necessitate a multi-faceted approach that includes promoting gender-equitable access to healthcare and nutrition interventions.

We found that 43.9% of under-five deaths occurred during the early neonatal period. Labour and the first 24 hours after birth present the highest vulnerability for the mother-baby dyad, with over four-fifths of neonatal deaths occurring during this period [40,41]. The primary causes of neonatal deaths are complications arising from prematurity, intrapartum asphyxia, and infections [42]. This highlights the critical importance of timely access to skilled birth attendants and emergency obstetric care, as delays in receiving appropriate interventions during labour and the immediate postpartum period can lead to preventable mortality. Strengthening referral systems and ensuring that health facilities are well-equipped to manage neonatal complications should be prioritised to improve survival rates. Skilled health workers and birth attendants who are adequately trained and regulated have been observed to contribute to a 16% reduction in neonatal deaths [5].

Our study findings indicate that 50% of households were located within a 30-minute proximity to the nearest health facility. Limited physical access to health facilities is significantly correlated with higher NMRs. Research has shown that for each kilometre increase in distance between households and the nearest health care facility, the risk of neonatal and child mortality rises by 15–20% [43,44]. This also suggests the existence not only if geographical barriers, but also infrastructural weaknesses, including inadequate transportation, poor road networks, and a lack of emergency medical services, all of which can exacerbate delays in care-seeking and provision of timely interventions.

Nearly 50% of mothers in our sample were married before the age of 20 and 60% had not received any formal education. Early marriage in developing countries is a major societal and health concern, leading to a 17% increase in the risk of mortality for children under five years of age [45,46]. Conversely, maternal education is strongly linked to reduced under-five mortality, with a discernible dose-response relationship. Children born to mothers who have completed secondary education (12 years of schooling) experience a 31% lower risk of mortality compared to those born to mothers with no education [47]. This underscores the intersection between social determinants of health and child survival, emphasising the need for policies that promote female education and delay early marriages. Educated mothers are more likely to access ANC, practice better hygiene, and seek medical attention promptly, all of which contribute to improved neonatal outcomes. Education also empowers women to make informed decisions and enhances their bargaining power, reducing the likelihood of early marriage and associated health risks [48,49].

Complications during pregnancy and childbirth are major causes of neonatal and under-five mortality in low- and middle-income countries (LMICs). However, timely and frequent ANC can mitigate these issues through early identification of danger signs, disease treatment, vaccination, nutritional supplementation, and counselling [50]. We found that 23% of the mothers of deceased neonates received no ANC and only 42% reported receiving four ANC visits. A pooled effect analysis indicates that ANC visits reduce under-five mortality by 34%, while specific studies in LMICs suggest mortality reductions ranging from 45% to 65% [50,51]. Therefore, adherence to the World Health Organization-recommended eight ANC contacts [52] is strongly recommended, especially in resource-constrained settings. In our study, significant maternal complications such as antepartum haemorrhage (15.7%), eclampsia/preeclampsia (25.8%), and maternal sepsis (9.9%) were reported among the mothers of deceased neonates. This suggests that ANC coverage alone is insufficient and must be coupled with improved quality of care, risk assessment, and timely management of complications to effectively reduce mortality.

Despite 90% of deliveries being attended by skilled birth attendants in our study, 30% of deliveries still occurred at home, where the absence of timely medical interventions increases risks. The high rate of labour complications (48.5%) among women delivering in health care facilities and the elevated NMR (60%) before hospital discharge highlights critical deficiencies in managing obstetric emergencies. Studies have shown that neonates delivered in a hospital setting have higher survival rates compared to those born at home, even when attended by skilled birth attendants. Home births in LMICs, often impeded by structural barriers to health care access, substantially elevate both total and early neonatal mortality (relative risk = 3.87) and are associated with other adverse neonatal outcomes [53], as most maternal and neonatal complications are preventable with proper medical care [54]. Our data illustrate the complex relationship between facility-based care and neonatal survival: while hospital deliveries were more common, early NMRs remained high, accounting for 82.1% of all neonatal deaths and 43.9% of the total under-five mortality. A possible explanation for the high proportion of neonatal deaths occurring in hospitals is the delay in seeking care, which diminishes survival chances, despite skilled attendance. Additionally, variations in the quality of hospital-based care, including suboptimal neonatal resuscitation, inadequate infection control measures, and insufficient postnatal monitoring, may further contribute to adverse neonatal outcomes.

The leading causes of neonatal death reported in the VASA interviews were birth asphyxia, preterm birth complications, and infectious diseases. This is consistent with a multi-country prospective cohort study conducted in eight African and South Asian countries, where birth asphyxia and sepsis accounted for approximately 70% of neonatal deaths [55,56]. Most common symptoms preceding neonatal deaths in our study included difficulty breathing, fever, and lethargy, which align with the known causes of neonatal mortality in our study. Furthermore, 21.2% of deceased neonates exhibited cyanosis at birth, indicating birth asphyxia. Despite high skilled birth attendance in the study, the persistence of asphyxia-related deaths suggests gaps in intrapartum monitoring, timely interventions, and neonatal resuscitation protocols. For decades, perinatal asphyxia has been one of the leading causes of neonatal mortality [55], with most intrapartum-related neonatal mortalities occurring due to birth asphyxia [57]. A verbal autopsy study in Pakistan and India supports these findings, identifying intrauterine hypoxia and infections as major contributors to neonatal mortality among preterm babies [58].

Most of the deceased neonates (82.3%) in our study were born preterm. Importantly, preterm birth is one of the leading causes of death among under-five children [59]. Premature infants are particularly vulnerable to complications, as they often develop fever due to infections or dehydration-induced hyperthermia and are ten times more likely to develop sepsis [60]. In low-income settings, half of the babies born at or before 32 weeks of gestation do not survive due to inadequate access to cost-effective interventions, such as the provision of warmth, breastfeeding support, and basic infection and breathing care; however, three-quarters of these deaths could be prevented with targeted and cost-effective interventions [61]. While cost-effective interventions such as kangaroo mother care (KMC), early breastfeeding, and basic neonatal infection control are known to reduce preterm mortality, their implementation in low-resource settings remains suboptimal.

The main causes of mortality among post-neonatal under-five children (1–59 months) were diarrheal diseases, pneumonia, other infectious diseases, injuries, and malnutrition. Most of the post-neonatal under-five mortality in LMICs, including South Asia, has been attributed to infectious diseases such as malaria, measles, diarrhoea, and lower respiratory diseases/pneumonia [59,62–65]. According to the World Health Organization, pneumonia, diarrhoea, malaria, and pre-term birth complications were the major causes of under-five mortality in 2020 [66]. The common symptoms reported in our study preceding the demise of under-five children included fever, lethargy, vomiting, sunken eyes, difficulty breathing, and diarrhoea, which align with the aforementioned causes. Despite improvements in sanitation, 30% of households lacked access to improved toilet facilities, emphasising the need for strengthened water, sanitation, and hygiene interventions. [67] Strengthening community-level interventions, including water, sanitation, and hygiene programmes and integrated child health services, remains critical for addressing these preventable deaths.

A subset of neonatal deaths could not be definitively attributed to specific causes. This may be due to inaccurate observation or recollection of symptoms by respondents. Additionally, certain rare conditions were not ascertained using the established methods due to their low prevalence or inadequate recall of symptoms by the mother, leading to these deaths being categorised as ‘unspecified’. Conversely, ‘undetermined’ causes refer to cases where, despite having sufficient information, the symptoms and circumstances did not align with any specific cause-of-death category, leaving the deaths unexplained, even after a thorough investigation [68]. In our study, the PCVA identified approximately 10.4% as ‘undetermined’. In the case of children aged 1–59 months, 7.4% of deaths were classified as ‘undetermined’, while 3.7% were deemed to be ‘unspecified’. While computer-based methods such as InterVA, InSilicoVA, and EAVA-1 offer standardised and scalable solutions, variations between computer-based methods are not uncommon, and are attributable to the inherent differences in their analytic approaches. These methods also tend to misclassify and generate a higher percentage of uncertain causes of death, particularly for complex or multi-causal deaths, which can result in an overrepresentation of undetermined causes of death, especially in neonates and young children [69–71]. The averaging approach we used here helped mitigate these discrepancies by smoothing the differences across methods, yielding results closer to the physician-coded causes, which remains one of the most reliable methods in many contexts. The PCVA leverages clinical expertise and provides more plausible causes of death, but is resource intensive. Studies have suggested that combining multiple VASA methods can improve accuracy by leveraging the strengths of each approach, a practice increasingly recommended in global health research [69,72]. Hybrid models integrating physician input with algorithmic methods could mitigate biases introduced by clinical coding behaviours and provide a more balanced estimation of CODs, particularly for complex deaths in resource-limited settings [73]. Although our primary aim was to report on causes of death, we also assessed inter-rater agreement, which showed moderate (satisfactory) consistency between physician reviewers. This highlights the inherent subjectivity in verbal autopsy interpretation and suggests the potential value of standardisation tools or automated methods to enhance consistency in future studies.

This study’s strength lies in its use of a population-based national survey and the VASA methodology to identify neonatal and child deaths within the community, extending beyond health facilities, thereby enhancing the generalisability of the findings to the national level. However, a significant limitation is our reliance on self-reported data, which may introduce recall bias in reporting birth outcomes and child deaths, resulting in possible under- or overestimation of mortality rates. Additionally, while we sought to mitigate classification bias in determining causes of death through verbal autopsies, the accuracy of these classifications depends on caregivers’ recall and interpretation, as well as the limitations of the verbal autopsy methods. Lastly, given the descriptive nature of our analysis, we did not adjust for potential confounders such as socioeconomic status, maternal comorbidities, and access to care; unmeasured confounding may therefore remain. Consequently, the contextual factors we describe should be interpreted as correlates rather than causal determinants of mortality, and the associations we discuss warrant confirmation in adjusted, analytic studies; our mortality and cause-of-death estimates, however, remain descriptive and are not affected by this limitation.

## CONCLUSIONS

Our findings highlight the persistent burden of neonatal and under-five mortality in Pakistan, emphasising the urgent need for targeted interventions. While no single VASA method consistently outperformed others across all causes of death, the combined use of PCVA and CCVA approaches strengthens cause-of-death attribution by leveraging their respective strengths. Despite methodological variations, all approaches consistently identified birth asphyxia, prematurity, and sepsis as the leading causes of neonatal deaths, while infectious diseases, malnutrition, and accidents were predominant among children aged 1–59 months. The high proportion of neonatal deaths among moderate-to-late preterm births underscores gaps in intrapartum and postnatal care, despite high skilled birth attendance and caesarean rates. Strengthening perinatal and neonatal intensive care, improving infectious disease control, and enhancing community-based health services, particularly in underserved areas, are critical to reducing child mortality and achieving sustainable improvements in child health outcomes. Future interventions should prioritise strengthening delivery room management, particularly for preterm and distressed newborns.

## Additional material


Online Supplementary Document
