# Supplementary material for: Rates and causes of neonatal and under-five mortality: insights from a national household survey in Pakistan
Source: J Glob Health. 2026 Sep 11;16:04211. doi: 10.7189/jogh.16.04211 (PMC13564848; doi:10.7189/jogh.16.04211)
Supplement: Supplementary file 1 [file jogh-16-04211-s001.pdf]

Supplement to: Malik AA, Arif S, Umer M, Nooruddin S, Habib MA, Rizvi A, Chauhadry IA, Soofi SB, Kalder HD, Black RE, Bhutta ZA. Rates and causes of neonatal and under-five mortality: insights from a national household survey in Pakistan. J Glob Health. 2026;16:04211.

Table S1. Distribution of neonatal and post-neonatal under-five deaths by age at death and sex in Pakistan

	Neonatal deaths, n (%)	Post-neonatal under-five deaths
**Age at death**		
0–3 days	1313 (63.3)	-
4–6 days	389 (18.8)	-
7–13 days	198 (9.5)	-
14–27 days	174 (8.4)	-
01–11 months	-	1042 (57.7)
12–59 months	-	764 (42.3)
**Sex**		
Male	1274 (61.4)	972 (53.8)
Female	800 (38.6)	834 (46.2)

Table S2. Household and maternal characteristics of neonatal and post-neonatal under-five deaths in Pakistan

	Neonatal deaths, n (%)	Post-neonatal under-five deaths
**Household characteristics**		
Household density (people/room), x̄ (SD)	3.8 (2.0)	3.9 (2.0)
Improved sources of drinking water	1898 (91.5)	1628 (90.1)
Improved toilet facilities	1456 (70.2)	1229 (68.1)
**Time taken to reach nearest health facility in hours**		
0–0.5	1079 (53.9)	884 (51.1)
0.6–1	567 (28.3)	517 (29.9)
>1 h	356 (17.8)	328 (19.0)
**Maternal age in years**		
<20	122 (6.5)	49 (3.0)
20–24	411 (21.8)	254 (15.7)
25–29	594 (31.4)	473 (29.2)
≥30	762 (40.3)	845 (52.1)
x̄ (SD)	28.1 (6.4)	29.7 (6.2)
**Mother's age at first marriage**		
≤15	214 (10.3)	174 (10.1)
16–20	1030 (49.7)	909 (52.8)
21–25	553 (26.7)	467 (27.1)
>25	180 (8.7)	171 (9.9)
First marriage age, x̄ (SD)	20.1 (4.2)	20.2 (4.2)

**Maternal Education**		
None	1185 (60.9)	1076 (65.1)
Primary	292 (15.0)	230 (13.9)
Secondary	330 (17.0)	244 (14.8)
Above secondary	139 (7.1)	104 (6.3)
Maternal education – completed years, x̄ (SD)	8.2 (3.4)	8.0 (3.6)
**ANC visits**		
1	131 (6.4)	-
2	297 (14.4)	-
3	300 (14.6)	-
≥4	861 (41.9)	-
None	468 (22.8)	-
**Skilled ANC provider**	1574 (97.0)	-
**Skilled birth attendant**	1870 (90.2)	-
**Premature birth**		
Yes	1966 (94.8)	1006 (96.5)*
No	87 (4.2)	36 (3.5)*
**Danger signs during pregnancy (as reported by respondent)**		
Vaginal bleeding	374 (18.0)	-
Severe headache with blurred vision	303 (14.6)	-
Severe abdominal pain	289 (13.9)	-
Broken water for 12 hours or more	240 (11.6)	-
Fever and too weak to get out of bed	235 (11.3)	-
Convulsions/fits	216 (10.4)	-
Fast or difficult breathing	182 (8.8)	-
Painful contractions every 20 min or less for 12 hours or more	157 (7.6)	-
Bloody, sticky discharge 12 hours or more	149 (7.2)	-
No immediate danger signs	1233 (59.5)	-
**Pregnancy complications in the last three months of pregnancy (as reported by respondent)**		
Maternal sepsis	103 (9.9)	-
Eclampsia/pre-eclampsia	268 (25.8)	-
Maternal anaemia	189 (18.2)	-
Maternal haemorrhage	163 (15.7)	-
Water broke six or more hours before labour	158 (15.2)	-
Diabetes	27 (2.6)	-

*Only for children 1–11 months of age.

Table S3. Place of delivery, characteristics of homebirths, and place of death for neonatal and post-neonatal under-five deaths

	Neonatal deaths, n (%)	Post-neonatal under-five deaths
**Delivery place**		
Home	622 (30.0)	394 (37.8)*
Hospital	1156 (55.8)	530 (50.9)*
Other facility	283 (13.7)	109 (10.5)*
**Women who reported having any complication during labour at health facility***		
Yes	698 (48.51)	-
No	741 (51.49)	-
**Tool used for cutting the cord for home deliveries**		
New from delivery kit/boiled razor blade	197 (31.0)	-
Old razor blade	141 (22.2)	-
Scissors	226 (35.6)	-
**Material used for tying the cord for home deliveries**		
New from delivery kit/boiled piece of thread	253 (39.8)	-
Unclean piece of thread	227 (35.7)	-
Cord clamp	63 (9.9)	-
**Cord care for all deliveries**		-
Alcohol/other antiseptic	325 (15.7)	-
Antibiotic ointment/ powder	303 (14.6)	-
Castor oil, mustard oil, or shea butter	255 (12.3)	-
Animal dung/mud/ash	15 (0.7)	-
**Place of death**		
Hospital (secondary or tertiary care level facility)	1146 (55.3)	633 (35.0)
Other health providers or facilities (primary care level)	48 (2.3)	16 (0.9)
En route to a health provider or facility	85 (4.1)	128 (7.1)
Home	793 (38.2)	987 (54.7)
Neonates who died before getting discharged from the delivery facility	413 (59.17)	-

*Convulsions, high blood pressure, excessive bleeding, premature labour, obstructed labour, prolonged labour, uterine rupture, and retained placenta.
